# Enhanced HPLC Method for Boar Taint Quantification

**DOI:** 10.1002/open.202300283

**Published:** 2024-05-29

**Authors:** R. Pereira‐Pinto, M. Barros, M. Vaz‐Velho, F. Mata, P. Pires

**Affiliations:** ^1^ CISAS – Centre for Research and Development in Agrifood Systems and Sustainability Instituto Politécnico de Viana do Castelo Rua Escola Industrial e Comercial de Nun'Álvares 4900-347 Viana do Castelo Portugal; ^2^ Escola Superior de Tecnologia e Gestão Instituto Politécnico de Viana do Castelo Avenida do Atlântico 644 4900-348 Viana do Castelo Portugal

**Keywords:** boar taint, androstenone, skatole, method validation, liquid chromatography

## Abstract

Boar taint is an unpleasant odour found in the carcasses of entire male pigs, resulting from androstenone and skatole accumulation during pubertal development, and impacting pork quality. This study proposes the validation of an adapted chromatographic method for quantifying skatole and androstenone in the pigs’ liquid fat using fluorescence detection. A good chromatographic separation was achieved, with skatole (SKA) and androstenone (AND) elution at 4.4 and 9.9 min., respectively. An external calibration method was applied, with calibration curves correlation coefficient of 0.9999 for both analytes. Detection limit values were 1.53 and 16.02 ng/g for SKA and AND, respectively. SKA recovery was 99.72±2.34 % (2.34 % RSD) and 102.84±1.62 % (1.57 % RSD) for AND. Results showed good precision values (repeatability <2.46 % RSD for SKA, <6.85 % RSD for AND; intermediate precision <2.87 % RSD for SKA, <6.98 % RSD for AND). The method‘s robustness was tested and the values were within the reference ranges. The validation results proved that the adaptation of an existing method resulted in good assessments of robustness, reliability and accuracy.

## Graphical abstract

This study validates an adapted chromatographic method for quantifying skatole and androstenone in pigs′ liquid fat using fluorescence detection, addressing the issue of boar taint in pork quality. Achieving good chromatographic separation, with skatole and androstenone elution at 4.4 and 9.9 min, respectively, the method demonstrates high precision and robustness, ensuring reliable and accurate assessments.

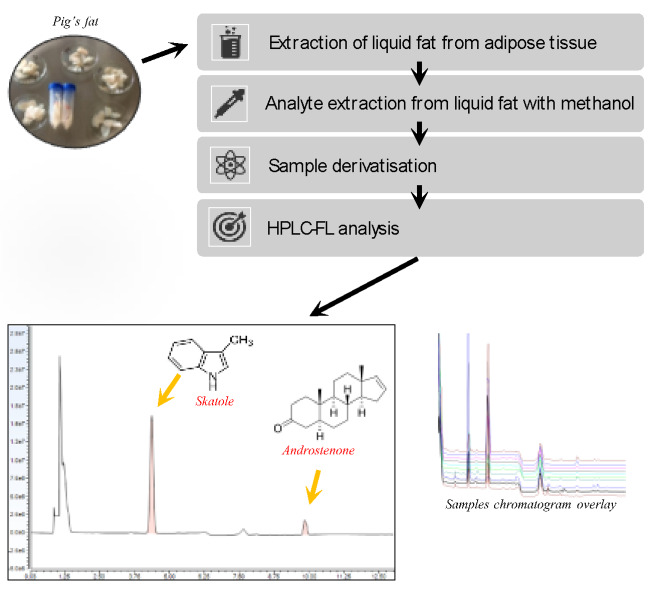

## 1 Introduction

The boar taint is an unpleasant odour found in the meat of 5 to 10 % of uncastrated male pigs[^1^, ^2^, ^3^, ^4^] (Aluwe et al., 2020; Baek et al., 1997; EFSA, 2004; Fredriksen et al., 2011) mainly caused by the accumulation of androstenone and skatole.^[5]^ As animals mature during puberty, these compounds build up in their adipose tissue,^[6]^ leading to the development of off‐odours and off‐flavours, creating an unfavourable perception of pork quality among consumers.

Detecting boar taint is vital for evaluating control strategies and selecting carcasses. Traditionally, boar taint analysis relied on skilled sensory panels, a subjective method where trained panellists smell and taste pork to detect taint. Despite rigorous training and statistical adjustments, the process remains intricate due to individual differences in the perception of skatole and androstenone.^[7]^ Chemical methods such as liquid or gas chromatography provide reliable and objective results for measuring boar taint, using chromatography to separate skatole (3‐methylindole) and androstenone (5α‐androst‐16‐en‐3‐one) based on their affinities to the stationary phase.[^8^, ^9^]

Hansen‐Moller^[10]^ first successfully determined both compounds simultaneously using high‐performance liquid chromatography (HPLC) with a fluorescence detector. Androstenone lacks native fluorescence, requiring prior derivatisation to enhance its detection.^[9]^. Several modifications of this method have been reported, optimising the procedure by simplifying extraction, reducing analysis time, and lowering detection limits.[^11^, ^12^, ^13^, ^14^, ^15^, ^16^] Due to limited inter‐laboratory comparison studies in boar taint detection, significant result bias exists between laboratories; therefore, further method harmonisation and standardisation are necessary to achieve accurate quantification of boar taint compounds.^[9]^ In pharmaceuticals, food, and cosmetics, quality control depends on well‐developed methods and testing tools, while accurate verification of analytical methods is essential for ensuring product quality and safety during use. Validation further confirms that these methods meet specific requirements with objective evidence.^[17]^

This study developed and validated a method for concurrently quantifying skatole and androstenone in pigs’ backfat using HPLC with fluorescence detection. This approach is based on a method previously described by Hansen‐Moller.^[10]^

## Experimental

### Reagents and Chemicals

The reference standards androstenone (5α‐androst‐16‐ene‐3‐one, CAS number 18339–16‐7) and skatole (3‐methylindole, CAS number 83‐34‐1) were obtained from Sigma Aldrich (St. Louis, MO, USA). Reagents were obtained from VWR International (Merck, Darmstadt, Germany) and were of analytical grade or HPLC grade. Dansylhydrazine and boron trifluoride‐methanol solution 14 % were obtained from Sigma Aldrich (St. Louis, MO, USA). Demineralised water was treated in a Milli‐Q Plus water purification system from Millipore (Bedford, MA, USA).

### Sample Preparation

Adipose tissue was collected from the backfat of the neck region of entire male pigs aged between 22 and 26 weeks (progeny of Large White × Landrace sows sired by Pietrain boars) after slaughter, on the premises of meat processing companies. Samples were vacuum packed and stored at −20 °C until analysis. Before conducting recovery studies, the samples underwent testing for skatole and androstenone contents to ensure they had low levels of these compounds.

### Sample Treatment

Fat extraction from adipose tissue was performed by melting the sample with microwave heating. The back fat was cut into thin flakes and placed in glass test tubes for 1 min at 800 W. After centrifugation for 15 min at 1100 *g*, a sample of approximately 1 mL of water‐free liquid fat was accurately weighed into Falcon tubes, and 1.0 mL of methanol:water (95 %) was added. The mixture was vortexed for 30 s, and tubes were then incubated for 10 min at 40 °C in an ultrasonic bath (Sonica® Ultrasonic Cleaner). Samples were centrifuged (JP Selecta Mixtasel) for 15 min at 1100 g and placed in an ice‐water bath for 10 min. After fat solidification, the supernatant was retrieved, placed in 1.5 mL Eppendorf tubes, and stored at 4 °C or frozen (−20 °C) if the sample was not analysed on the same day.

### Derivatisation

Derivatisation principles described by Hansen‐Moller^[10]^ were followed, applying dansylhydrazine in 2‐fold stoichiometric proportions. Samples were manually derivatised at room temperature by sequentially adding 75 μL of dansylhidrazine (0.1 % v/v in methanol), 50 μL of deionised water, 40 μL of BF_3_ 14 % solution in methanol, and 500 μL of treated sample in an Eppendorf tube, shaking for 10 s and waiting 5 min before injection.

### High‐Performance Liquid Chromatography System

An HPLC system (Thermo Scientific Dionex UltiMate 3000, Waltham, MA, USA) equipped with a quaternary pump, a column oven, a fluorescence detector and a Kromasil 100‐5‐C18 5 μm, 250×4.6 mm column (AkzoNobel, Bohus, Sweden) operating at 40 °C were used. The composition of mobile phase eluents was as follows: (A) acetic acid 0,1 % (v/v), (B) acetonitrile; (C) tetrahydrofuran, (D) methanol‐water 95 % (v/v), with the following gradient profile: 0.0‐5.0 min: 45 % A, 55 % B; 5.0–6.0 min: 40 % A, 55 % B, 5 % C; 6.0–6.1 min: 20 % A, 30 % B, 30 % C, 20 % D; 6.1–12.0 min: 40 % B, 40 % C, 20 % D; 12.0–13.0 min: 45 % A, 55 % B. Fluorescence detection was performed with excitation at 285 nm, emission at 340 nm (0–6.0 min), followed by excitation at 346 nm and emission at 521 nm (6.1–13.0 min), with data collection rate of 25.0 Hz. In all assays, 20 μL of the sample was injected. Chromatograms and integration were achieved using Thermo Scientific Dionex Chromeleon version 7.2 SR5 (Thermo Scientific, Waltham, MA, USA).

### Method Validation

The proposed method was validated for identity, specificity, linearity, range, accuracy, precision, robustness, and system suitability.

#### Identity and Specificity

The identity confirmation for skatole and androstenone was obtained by comparing the retention times of the calibration solutions with those of control materials and test samples.

#### Linearity and Range

For the construction of calibration curves, skatole and androstenone stock solutions were prepared in methanol:water (95 % v/v). Working solutions were prepared by further dilution of the stock with methanol:water (95 % v/v). Dilutions containing both skatole and androstenone standards were prepared from standard solutions. After derivatisation, each dilution was injected into the HPLC in duplicate, and the average peak areas were then calculated; the calibration curves were constructed, and the regression equations were also computed.

#### Accuracy and Precision

The method‘s accuracy, expressed in recovery studies, was performed by the standard addition method by adding known amounts of standard analytes to liquid fat. Due to the unavailability of certified reference material, recovery was evaluated by adding known quantities of skatole and androstenone to liquid fat samples with low background concentrations. To evaluate the method‘s precision, repeatability and within‐laboratory reproducibility were determined. Both validation parameters were assessed by calculating the relative standard deviations. Repeatability was investigated by injecting three concentration levels for skatole and androstenone, using three replicate determinations for each concentration. The inter‐day precision for the proposed method was studied by following the same procedure for assessing repeatability on three non‐consecutive days.

#### Robustness

The robustness of the developed method was assessed by modifying the flow rate and the mobile phase composition to observe any deviations from the optimised chromatographic condition.

#### System Suitability

System suitability was assessed by determining parameters such as peak asymmetry, number of theoretical plates and height equivalent to theoretical plate.

## 2 Results and Discussion

Following the experiment developed by Hansen‐Moller,^[10]^ fluorescence was opted for detection due to the favourable fluorescent characteristics of skatole, offering the advantage of easily derivatising androstenone with dansylhydrazine, resulting in enhanced selectivity and sensitivity. During the initial tests of the present study, it was noticed that the amount of dansylhydrazine suggested by Hansen‐Moller^[10]^ for sample derivatisation (30 μL of 2 % dansylhydrazine to 140 μL of the sample) resulted in excessive fluorescence, leading to chromatograms with saturated peaks and significant interference. Dansylhydrazine derivatisation reagent interferences were encountered in determining the androstenone levels in fat when using a high molar ratio. During the method modification, a reduction in this reagent was studied, and other derivatisation conditions were explored, including temperature variations (room temperature, 40 °C, 60 °C) and derivatisation time (1, 5, 15, and 30 min). The results obtained during the test phase of the optimal derivatisation conditions and identification of the retention times of the compounds allowed to conclude that at room temperature, with a reaction time of 5 min and using 75 μL of 0.1 % dansylhydrazine (a reduction very significant in relation to the method proposed by Hansen‐Moller^[10]^), 50 μL of deionised water and 40 μL of BF_3_‐methanol solution in 500 μL of the sample, the chromatograms exhibited minimal interference, and the androstenone peak demonstrated improved definition. However, for reaction times exceeding 5 min, the peaks tended to broaden, and the tested temperature variations for 5 min did not lead to discernible differences in the chromatograms.

**Figure 1:**
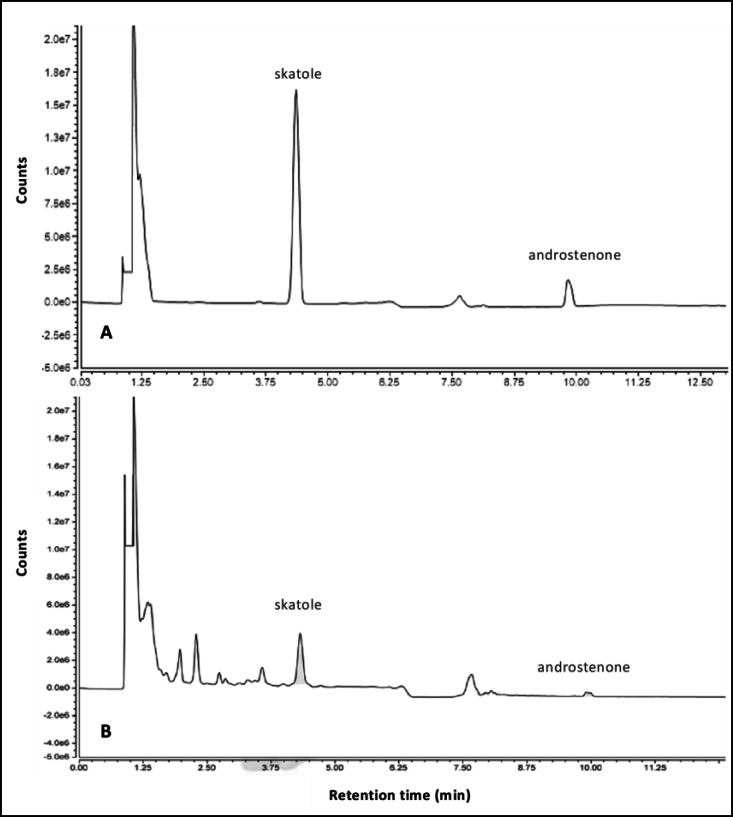
Chromatograms of the standards solution (A) and fat sample (B).

To confirm the identity of the compounds, skatole and androstenone standard solutions (see Reagents and Chemicals section) were injected in order to check retention times (Figure 1). Subsequently, solutions of fat samples were injected to confirm the presence of the analytes. In all cases, the retention times were 4.3 min for skatole and 9.9 min for androstenone. The method‘s specificity is also demonstrated in Figure 1, where skatole and androstenone are completely separated. Based on the obtained data, it can be concluded that skatole and androstenone can be simultaneously determined without interference.

**Figure 2:**
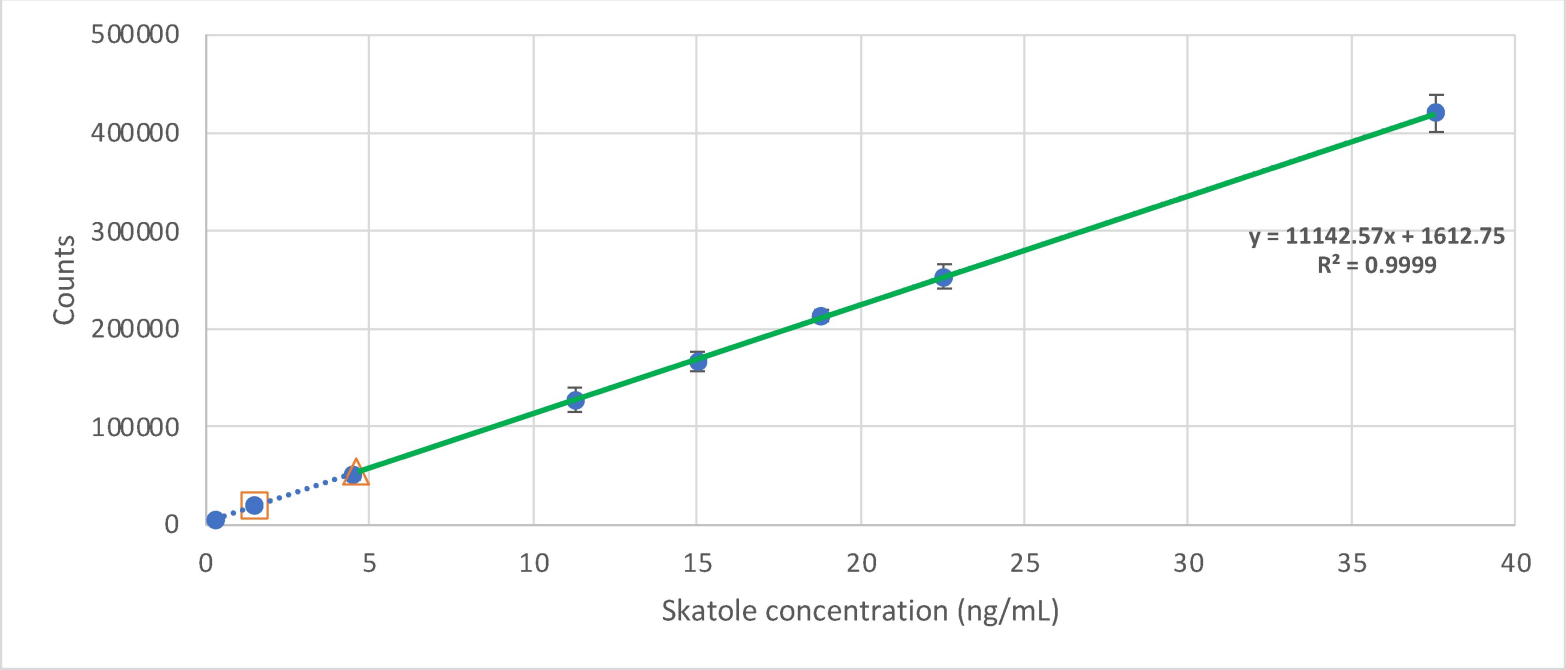
Graphical representation of the calibration curve for skatole in standard solutions. The squared plot represents the calculated Limit of Detection and the triangle plot the Limit of Quantification. The green line highlights the working range.

**Figure 3:**
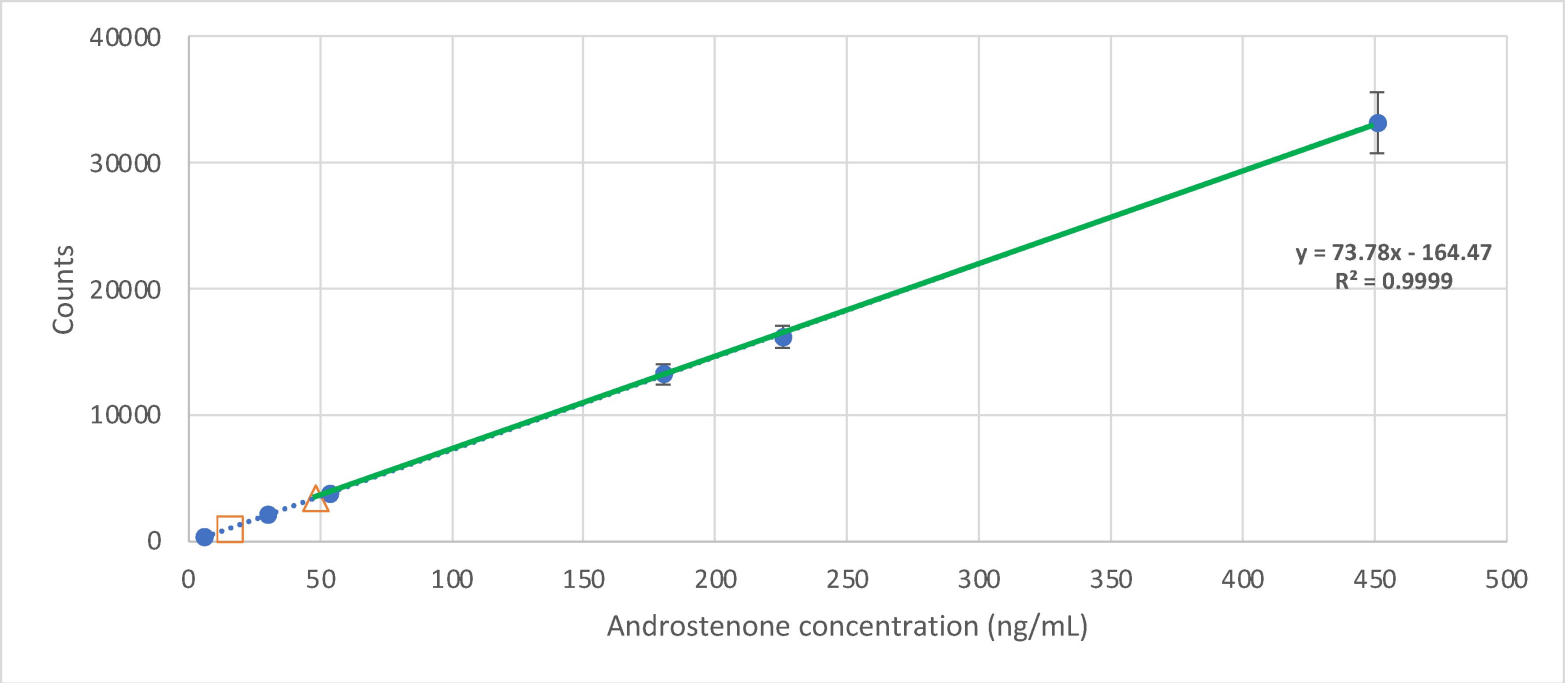
Graphical representation of the calibration curve for androstenone in standard solutions. The squared plot represents the calculated Limit of Detection and the triangle plot the Limit of Quantification. The green line highlights the working range.

The linearity of peak area responses *versus* concentrations was demonstrated by linear least square regression analysis, and data are shown in Table 1. The linear regression equations were y=11142x+1612.8 (r=0.999) for skatole (Figure 2) and y=73.777x–164.47 (r^2^=0.999) for androstenone (Figure 3), where y is the peak area of standard solution and x is the analyte concentration. The linearity of the method was evaluated by analysing different concentrations of the analytes. According to Magnunsson & Ornemark^[18]^, at least six concentrations should be used. In this study, eight concentrations were selected in the range of 0.3 to 37.6 ng/mL for skatole and six concentrations in the range of 6.0 to 451.1 ng/mL for androstenone. During calibration studies, there was a need to adapt to a range of values that are commonly found, and according to the available literature, there is great variability. This dispersion is typical when skatole and androstenone are measured in the pigs’ population, as reported in similar studies.[^10^, ^11^, ^19^, ^20^, ^21^] For instance, Prusa et al.^[14]^ conducted a study to determine the prevalence of skatole and androstenone in the fat of pigs in the United States, analysing 600 samples. The mean concentrations for skatole ranged from 18.7 ng/g (limit of quantification) to 252.4 ng/g, while for androstenone, they varied from 200 ng/g (limit of quantification) to 3412 ng/g. Similarly, Walstra et al.^[22]^ investigated skatole and androstenone levels in male and female pigs across six European countries (Spain, France, Netherlands, United Kingdom, Denmark, and Sweden). On average, skatole and androstenone values were reported to be 70 ng/g and 30 ng/g, respectively. For entire males, skatole averages ranged from 100 ng/g to 170 ng/g, while androstenone levels varied between 800 ng/g and 1270 ng/g. Similar studies also present a wide range of studied concentrations, as presented in Table 2.

**Table 1:** Results of validation parameters of the proposed HPLC method for skatole and androstenone analysis.

	Skatole	Androstenone
Working range (ng/mL)	4.64–37.6	48.54–451.1
Slope	11142,57	73.78
Intercept	1612.75	−164.47
Correlation coefficient (r)	0.9999	0.9999
LoD (ng/mL)^a^	1.53	16.02
LoQ (ng/mL)^a^	4.64	48.54
Recovery %	99.72±2.34	102.84±1.62
RSD %^b^	2.34	1.57

LoD – Limit of Detection; LoQ – Limit of Quantification ^a^ Calculated according to the following equations LoD=3.3 s/S and LoQ=10 s/S, where s is the standard deviation of the response, and S is the slope of the calibration graph. ^b^ Relative standard deviations (RSD) (n=3) of sample recovery concentrations.

**Table 2:** Values of LoD and ranges of used standard concentrations for skatole and androstenone simultaneous determination found in the literature.

Author	Skatole (ng/mL)	Androstenone (ng/mL)	Ranges of standard concentrations (ng/mL)	Instrumental technique
Hansen‐Moller^[10]^	<3	20	SKA: 1–1000 AND: 10–5000	HPLC‐FL
Aaslyng et al.^[11]^	9.9*	20	Not reported	HPLC‐FL
Pinto et al.^[13]^	5	53	SKA: 1.6‐63.0 AND: 38–1504	HPLC‐FL
Fischer et al.^[23]^	0,1	35	SKA: 0.5‐1000 AND: 50–5000	HS‐SPME‐GC/MS
Verheyden et al.^[24]^	16.5*	41.3*	SKA: 50–1600 AND: 125–2000	LC–MS
Buttinger et al.^[25]^	2.8	14.9	SKA: 50–1000 AND: 100–5000	LC–MS
Wauters et al.^[26]^	0.5	1.0	SKA:0.5‐200 AND:0.5‐200	UHPLC‐HR‐Orbitrap MS
Bekaert et al.^[27]^	2.5	7	SKA: 100–2000 AND: 100–2000	UHPLC‐HR‐Orbitrap MS

* The calculated value is based on the quantification limit mentioned in the study. SKA‐skatole; AND‐androstenone.

External standard calibration plots analyte response *versus* concentration in chromatographic tests, however it is susceptible to volume errors or underestimating sample preparation losses. In order to accurately determine the analyte concentration in samples, internal standard calibration entails introducing a carefully selected molecule to each sample. This gives response ratios against their respective amounts.^[28]^ Several methods for simultaneous quantification of skatole and androstenone use internal standard calibration. The most commonly used internal standards are 2‐methylindole and androstanone for skatole and androstenone detection, respectively.[^10^, ^11^, ^12^, ^16^] During the initial assessments of retention times and the identification of skatole, 2‐methylindole was evaluated as an internal standard. However, due to the proximity of retention times, the skatole‘s peak resolution was compromised, even though the peaks did not co‐elute. Consequently, the external standard calibration method was chosen as an alternative.

The primary objective of determining the Limit of Detection (LoD) is generally to identify the minimum concentration of the analyte in a sample that can be reliably detected, utilising a specific measurement procedure and a predetermined level of confidence. The Limit of Quantification (LoQ) represents the minimum analyte concentration that can be reliably determined with acceptable performance, which may be defined differently by various guidelines, considering factors such as precision, trueness, or measurement uncertainty.^[18]^ This study compared the LoD values for skatole and androstenone (Table 1) with those obtained in published studies (Table 2). The comparison with other methods using the same equipment confirms that the proposed method has lower detection limits, making it better suited for detecting smaller amounts of the analyte. Nevertheless, higher performance and more accurate results are often achieved with more sophisticated and costly equipment, such as mass spectrum chromatography.

The recovery was assessed using the standard addition method, where known amounts of standard analytes were added to the liquid fat. Three replicates of liquid fat samples with known concentrations of skatole and androstenone were spiked with 38.6, 19.3 and 4.8 ng/mL of skatole and 442.0, 88.4 and 44.2 ng/mL of androstenone. These concentrations were selected based on the values within the tested linearity range. The obtained recovery values are shown in Table 1, 99.72 % for skatole and 102.84 % for androstenone. According to EC‐2017^[29]^, acceptable mean recoveries for initial validation are within the range of 70–120 %.

The assay‘s precision was evaluated by measuring both repeatability and intermediate precision. As per Magnunsson & Ornemark,^[18]^ repeatability is a measure of the variation in results when a single analyst uses the same equipment to perform measurements over a short timescale, and it is expected to yield the slightest variation in results. The investigation of repeatability involved injecting three different concentration levels of skatole and androstenone, with three replicates for each concentration. To assess the inter‐day precision of the proposed method, the same procedure for repeatability was followed on three non‐consecutive days. Table 3 presents the results obtained from these determinations, expressed in relative standard deviation (RSD). For skatole, the RSD values of both repeatability and intermediate precision tend to increase as the analyte concentration decreases, ranging between 1.23 % and 2.87 %. However, the differences between intra‐day and inter‐day are not statistically significant (p>0.05). As for androstenone, the RSD values for precision are higher compared to skatole, ranging between 5.28 % and 6.98 %. However, it does not tend to be higher with lower analyte concentrations. Also, no statistically significant differences (p>0.05) were observed between within‐day and intra‐day deviations.

**Table 3:** Results of precision parameters for inter‐day assays.

	Skatole			Androstenone		
Analyte concentration (ng/mL)	80	40	10	650	300	175
Repeatability (% RSD)	1.23	1.78	2.46	6.21	5.28	6.85
Intermediate precision (% RSD)	1.33	1.88	2.87	6.98	5.75	6.88
ANOVA *p‐value*	0.16	0.21	0.07	0.11	0.15	0.40

RSD – Relative standard deviation.

During the development phase, it is crucial to consider the assessment of robustness, which varies depending on the specific procedure being studied.^[30]^ The robustness of an analytical approach is a measure of its capacity to remain unaffected by small variations in method parameters and indicates its reliability during normal usage.^[18]^ The proposed method tested robustness by studying the effect of changing the flow rate to 0.1 mL/min and changing the mobile phase by adding or subtracting 5 % of acetonitrile throughout the run (Table 4). When the flow rate was modified, there were variations in retention times. As expected, a decrease in flow rate resulted in a slight increase in retention time (Rt) for skatole and androstenone, with a difference of approximately 0.3 and 0.2 min, respectively. Conversely, an increased flow rate led to an earlier Rt of 0.1 min for both analytes. However, these changes were found to be insignificant. Similarly, the Rt values were also affected when there was a change in the mobile phase, specifically by altering the eluent used throughout the run. Decreasing the solvent concentration caused a delay in Rt, with a difference of 1.4 min for skatole and 0.9 min for androstenone. On the other hand, increasing the solvent concentration by 5 % resulted in an earlier Rt, with a difference of 0.4 min for skatole and 0.5 min for androstenone. The theoretical plate number is a measure of column efficiency, that is, how many peaks can be located per unit runtime of the chromatogram. According to CDER‐1994,^[31]^ the theoretical plate number (N) depends on elution time but, in general, should be >2000. In the tested conditions, the N was above that value (Table 4). Also, the peak asymmetry, or tailing factor (T), was less than 2, the value recommended as acceptable by the previously mentioned source.

**Table 4:** Results of “Robustness” testing of the proposed HPLC method.

Conditions	*Rt*		*N*		*T*	
	SKA	AND	SKA	AND	SKA	AND
Flow rate						
1.9 mL/min	4.6	10.1	3973	23436	1.10	1.15
2.1 mL/min	4.2	9.8	3206	22042	1.24	1.08
Mobile phase composition						
‐5 % ACN	5.7	10.8	4755	19803	0.98	1.33
+5 % ACN	3.9	9.4	5836	28451	0.96	1.16

SKA – skatole; AND – androstenone; Rt – retention times, in min; N – number of theoretical plates; T – tailing factor; ACN – acetonitrile.

The System Suitability Test is typically conducted to assess the adequacy and effectiveness of the entire chromatographic system, not only before its initial use but also throughout the analysis. The term “system suitability” is frequently misused and often used interchangeably with calibration and validation. In reality, system suitability serves as a tool to verify that the entire analytical system functions correctly at a specific moment.^[32]^ The System Suitability was assessed for the proposed HPLC method (Table 5). According to the Center for Drug Evaluation and Research guidelines^[31]^ the obtained data from the chromatographic system demonstrate that it is adequate for analysis.

**Table 5:** System Suitability Parameters of the proposed HPLC method.

Parameters	SKA	AND	Reference value
Asymmetry	1.04	1.19	T <2
Number of theoretical plates (N)	4067	22966	N >2000
Height equivalent to theoretical plate (HETP, in millimetres)	0.06	0.01	Smaller values mean higher column efficiency

SKA – skatole; AND – androstenone.

## 3 Conclusions

The requirement to precisely measure the substances that cause boar taint prompts a reevaluation of current approaches and their adaptation to the technology at hand. The outcomes of this method allow for the simultaneous detection of skatole and androstenone to occur quite quickly, with low detection and quantification limits, and with relatively straightforward sample preparation. The validation results show that the approach is accurate and reliable, with good analyte recovery rates that are appropriate for use in extensive laboratory research examining methods to reduce boar taint.

## Funding

This work was financially supported by Project TECH ‐ Technology, Environment, Creativity and Health, Norte‐01‐0145‐FEDER‐000043, supported by Norte Portugal Regional Operational Program (NORTE 2020), under the PORTUGAL 2020 Partnership Agreement, through the European Regional Development Fund (ERDF).

## Conflict of Interests

The authors declare no conflict of interest.

4

## Data Availability

The data that support the findings of this study are available from the corresponding author upon reasonable request.
